# Low-Energy Pulsed Ion Beam Technology with Ultra-High Material Removal Resolution and Widely Adjustable Removal Efficiency

**DOI:** 10.3390/mi12111370

**Published:** 2021-11-08

**Authors:** Guangqi Zhou, Ye Tian, Feng Shi, Ci Song, Guipeng Tie, Gang Zhou, Lingbo Xie, Jianda Shao, Zhouling Wu

**Affiliations:** 1 College of Intelligence Science and Technology, National University of Defense Technology, Changsha 410073, China; guangqizhou@foxmail.com (G.Z.); sf.wind@yahoo.com (F.S.); sunicris@163.com (C.S.); tieguipeng@163.com (G.T.); zg2206553079@foxmail.com (G.Z.); lingbotse@163.com (L.X.); 2 Hunan Key Laboratory of Ultra-Precision Machining Technology, Changsha 410073, China; 3 Laboratory of Science and Technology on Integrated Logistics Support, National University of Defense Technology, Changsha 410073, China; 4 Laboratory of Thin Film Optics, Shanghai Institute of Optics and Fine Mechanics, Chinese Academy of Sciences, Shanghai 201800, China; jdshao@siom.ac.cn; 5 Key Laboratory of Nondestructive Testing for Super Smooth Surface of Anhui Province, Hefei 230031, China; zlwu@zc-hightech.com

**Keywords:** low-energy pulsed ion beam, pulse frequency, pulse duty cycle, ultra-high removal resolution

## Abstract

High-precision optical component manufacturing by ion beam machining tools with ultra-high material removal resolution and dynamically adjustable removal efficiency is important in various industries. In this paper, we propose a low-energy pulsed ion beam (LPIB) technology that can obtain a single pulse with high-resolution material removal by adjusting the pulse frequency and duty cycle, and enable the dynamic adjustment of the removal efficiency. The pulse frequency is 1–100 Hz, and the duty cycle is 0–100%. For monocrystalline silicon, the pulse frequency and duty cycle are set to 100 Hz and 1%, respectively; thus, the single-shot pulse depth removal resolution of material is 6.7 × 10^−4^ nm, which means every 21 pulses can remove one silicon atom layer. Compared with IBF, where the removal resolution of the maximum depth is about 0.01 nm, the controllable resolution is one to two orders of magnitude higher. There is a linear relationship between the removal efficiency of the pulsed ion beam removal function and the pulse duty ratio. The material removal of a single pulse can be adjusted in real time by adjusting the pulse duty cycle and frequency. Owing to its high resolution and wide adjustable removal efficiency, LPIB has broad application prospects in the field of sub-nano-precision surface modification, quality tuning of inertial resonant devices, and so on. This technology is expected to advance surface processing and ultra-precision manufacturing.

## 1. Introduction

To obtain the desired ultra-high precision or special physical and chemical properties, researchers must use various precision material removal technologies to explore the nano world. They can accurately obtain the required precision and mass distribution on various spatial scales [1,2,3,4]. The tool foundation and technical premise of material and manufacturing technology’s progress is advanced precision processing technology. Based on the non-contact removal mechanism and controllability of material sputtering, low-energy ion beams (single ion energy of less than 2 keV [5]) have been widely used in ultra-precision manufacturing and in the preparation of special physical and chemical states [6,7,8,9,10,11]. For example, the focused ion beam (FIB) has become a necessary tool to explore the atomic world by accurately removing atomic materials [12,13]. In addition, IBF is used to fabricate optical surfaces, and has sub-nanometer precision [14,15,16,17,18].

However, FIB can only process micro zones as its beam diameter is in the micrometer level, and its volume removal efficiency is correspondingly low [19]. The removal efficiency of IBF is non-adjustable in real time. Due to the removal resolution of IBF being insufficient, an additional removal layer is necessary during processing. For normal continuous ion beam technologies, researchers have tried to achieve higher removal resolution using a smaller beam diameter and shorter sputtering time. However, this method is limited: a smaller diameter directly reduces the removal speed. A shorter sputtering time needs better dynamic performance of motion parts, preventing its further application.

**Figure 1:**
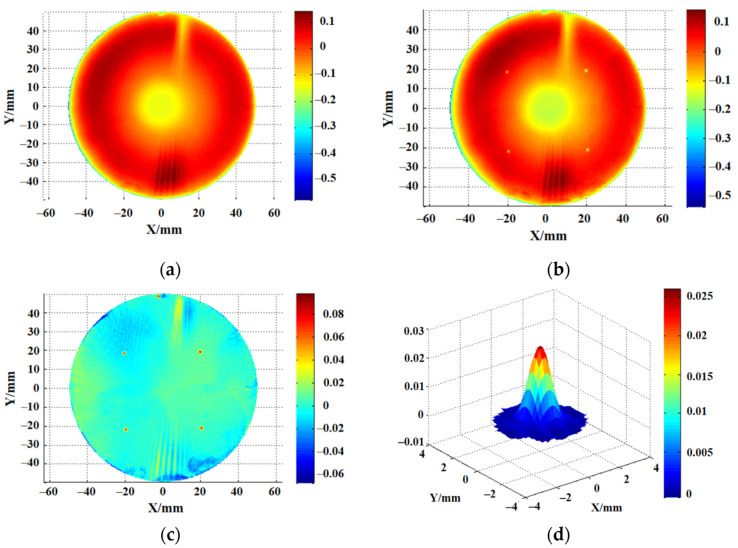
Material removal resolution of IBF: (**a**) the initial surface; (**b**) removed surface; (**c**) surface subtraction; (**d**) 3.3 mm removal function.

For normal IBF technology, we obtained depth removal resolution through experiments. The time resolution of the machine tool used in the experiment was about 0.02 s. We carried out a fixed-point material removal experiment on a single-crystal silicon sample to obtain the removal function, as shown in Figure 1.

We used a wave interferometer to measure the surface data before and after processing. The material removal amount can be determined by subtracting the surface data before and after processing. Figure 1 shows the experimental process of determining the IBF removal resolution. Figure 1a shows the surface data before processing, Figure 1b shows the surface after processing, and Figure 1c shows the surface after subtraction. The removal function was obtained by calculating the material removal amount and dwell time. Figure 1d shows a 3.3 mm removal function achieved with a corresponding material peak removal rate (PRR) of 0.041 μm/min. PRR represents the maximum removal depth in per unit time. So, IBF depth removal resolution can be obtained from Equation (1):

(1) Rev=PRR⋅Δt

where the Rev is the depth removal resolution, PRR is the peak removal rate, and the Δt is the time resolution of the machine tool. So, the removal resolution of the depth was about 0.01 nm on the monocrystalline silicon by controlling the acceleration and local dwell time. Furthermore, the removal rate must remain stable and cannot be adjusted in real time. Therefore, a removal function with higher resolution cannot be used in actual processing processes due to its extremely low removal efficiency. It does not seem as if there is a direct removal and non-contact surface material treatment method that can simultaneously achieve micro ultra-high controllable removal resolution and adjustable removal efficiency [20].

Combining the advantages of the laser pulse’s precise control and the non-contact removal of the low-energy ion beam figuring, this paper proposes a low-energy pulsed ion beam (LPIB) removal method. The continuous ion beam is transferred into a pulsed beam, and a single pulsed ion beam can be obtained by controlling the frequency and duty cycle to accurately obtain different material removal rates. The pulse duty cycle is continuously adjustable from 0% to 100%, and the pulse frequency is continuously adjustable from 1 to 100 Hz. By reducing the pulse duty cycle and increasing the pulse frequency, a single pulse with ultra-high material removal resolution can be obtained. Simultaneously, a single pulsed ion beam with higher material removal efficiency can also be obtained by increasing the pulse duty cycle.

In Section 2, according to the theory of ion beam sputtering, this paper presents using pulse voltage to extract the ion beam and establish the removal model after introducing pulse voltage. In Section 3, the experiments on the depth removal resolution of LPIB and the relationship between the removal rate and duty cycle are completed. Furthermore, the stability of LPIB is verified.

The results indicate that LPIB has good stability, predictability, and linearity. The depth removal resolution is 6.7 × 10^−4^ nm, which is one to two orders of magnitude higher than that of IBF. The removal rate varies linearly with the duty cycle.

Overall, LPIB is a material processing technology with ultra-high removal resolution and wide adjustable removal efficiency. It has various applications in the fields of inertial resonance device quality modification, sub-nanometer precision surface modification, micro-nano electromechanical systems, special surface preparation, and so on. It will promote the progress of material processing technology and ultra-precision manufacturing technology.

## 2. Theoretical Background

LPIB removes material by ion sputtering, just like FIB. LPIB inherits all the advantages from IBF. Its removal efficiency can be expressed by sputtering yield, which is the ratio of sputtered material atoms and incident ions. The sputtering yield is given as:

(2) $$Y=\frac{n_{o}}{n_{i}}$$

where the $n_{o}$ is the number of atoms sputtered from the element and $n_{i}$ is the number of incident ions. According to Sigmund sputtering theory [21,22], the number of material atoms sputtered out can be controlled by proportionally changing the number of incident ions [23].

For the same material, keeping the other parameters constant, the etching rate has a linear relationship with the ion density. By precisely controlling the amount of incident ions, the corresponding amount of material removal can be obtained.

This paper introduces using pulse voltage to extract the ion beam, and the continuous ion beam is converted into a pulsed ion beam. As long as the pulse voltage is precisely controlled, we can produce a controllable pulsed ion beam.

**Figure 2:**
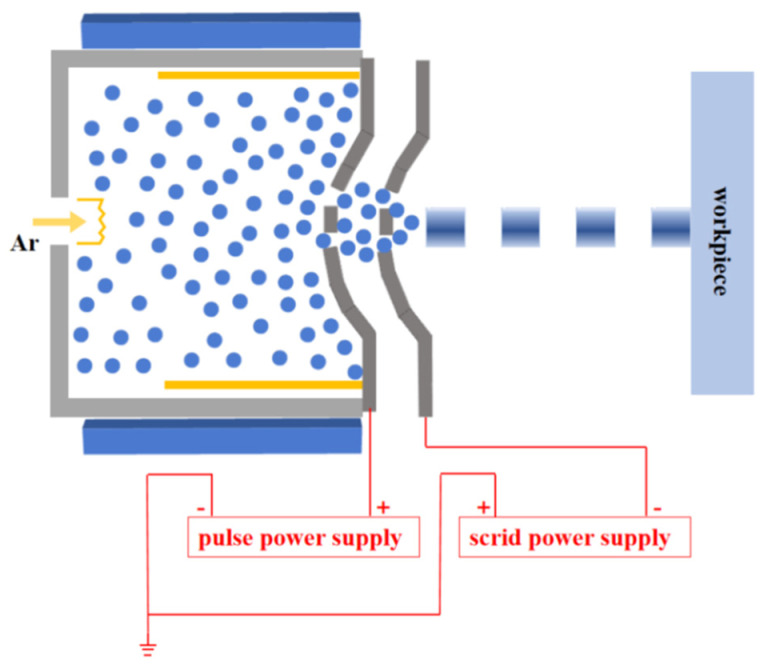
Principle diagram of pulsed ion beam extraction.

As shown in Figure 2, using pulse voltage to extract the ion beam, the continuous ion beam is transferred into a pulsed beam. We define the ratio of beam extraction in a cycle as the duty cycle. The duty cycle and frequency of the pulsed ion beam are related to the pulse voltage. LPIB controls the removal material of a single pulse by controlling the pulse frequency and duty cycle. This makes it easier to achieve higher material removal resolution and improve removal efficiency. As long as the pulse voltage can be accurately controlled, the corresponding pulsed ion beam can be obtained.

## 3. Experiment Details and Data Analysis

### 3.1. Ultra-High Material Removal Resolution

**Figure 3:**
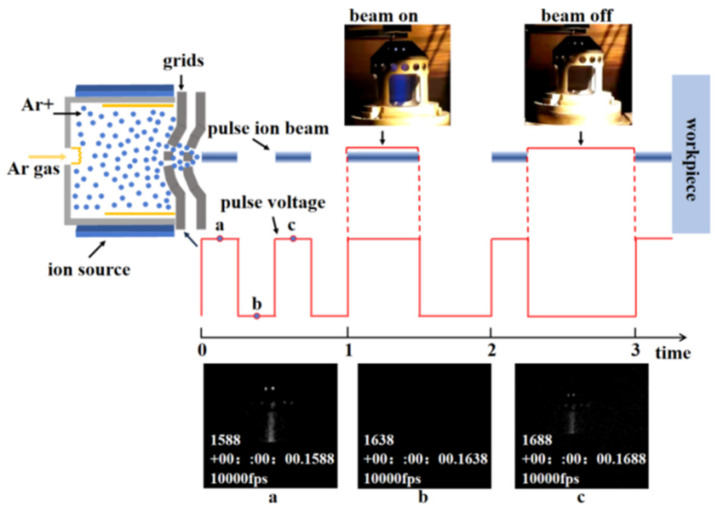
Principle diagram and experimental pictures of the LPIB.

By changing the structure and control mode of an ion optics system, an LPIB polishing tool was obtained, which has adjustable frequency and duty cycle. The related pulse phenomena are presented in Figure 3.

As shown in Figure 3, the 100 Hz pulse processing was recorded by a high-speed camera called FASTCAM Nova S12 (Photron, Tokyo, Japan). The pulsed ion beam changed with the pulse voltage. When the pulse voltage was at low potential, the ion beam was not extracted. The common equipment parameters are presented in Table 1.

In this study, the pulse voltage was changed to dynamically control the ion beam extraction. By precisely controlling the number of ion pulses, ultra-high material removal resolution was achieved. During the experiment, the duty cycle of the pulse was kept at 1%, and the pulse frequency was kept at 100 Hz. The pulse numbers increased linearly from 12,000 to 48,000.

**Figure 4:**
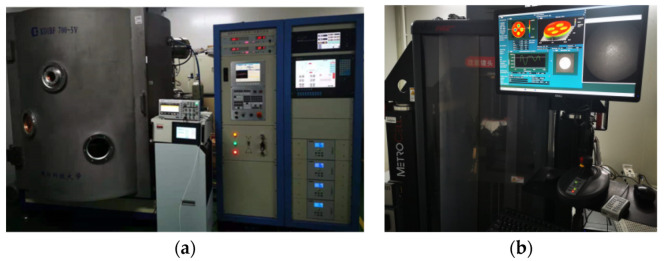
Experimental equipment: (**a**) processing equipment; (**b**) measuring equipment.

Figure 4a shows the processing equipment: KDIBF 700-5V (National University of Defense Technology, Changsha, China). As shown in Figure 4b, the surface data were measured by a Zygo VeriFire Asphere (Zygo, Middlefield, CT, USA). The removal depth and the spatial distribution of material removal were obtained by subtracting the surface data before and after pulsed ion beam figuring.

**Figure 5:**
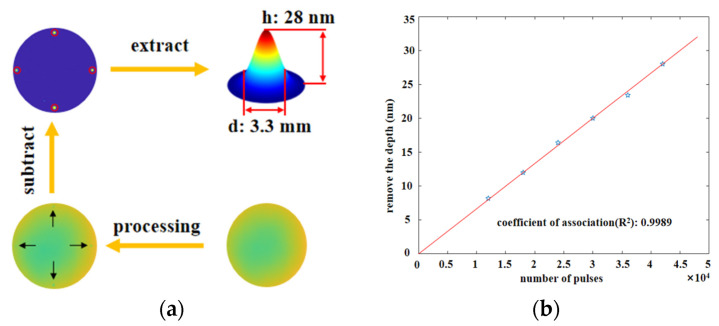
Material removal resolution: (**a**) extraction process of removal amount; (**b**) linearity between the material removal and pulse number.

Figure 5a shows the data extraction process: we obtained the material removal amount by subtracting the shape after processing from that beforehand. According to the material removal amount and the dwell time, the removal function was obtained. The removal depth can be obtained by the multiplying peak removal rate and dwell time. The results show that the peak removal rate of the corresponding materials was 0.0040 μm/min. Figure 5b shows that the removal depth changed linearly with the number of pulses, and the linear correlation coefficient (R^2^) reached 0.9989. By calculating the experimental results, the obtained depth resolution of the single-pulse removal of monocrystalline silicon material was 6.7 × 10^−4^ nm. Compared to IBF, a higher order of magnitude material removal resolution was achieved by using pulse control instead of motion axis parameter control.

### 3.2. Verification of the Linear Relationship between Duty Cycle and Material Removal

To verify the linear relationship between the pulse duty cycle and material removal, fixed-point material removal was conducted for 6 min using different duty cycles, and the material removal was measured.

**Figure 6:**
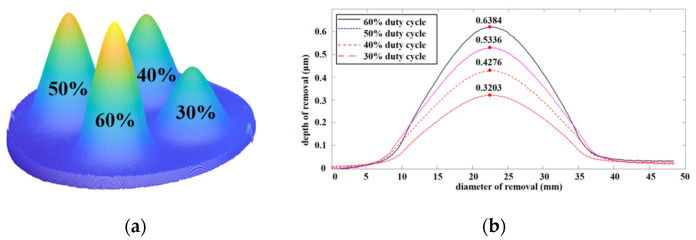
Removal models for different duty cycles: (**a**) material removal model; (**b**) removal depth curves of different duty cycles.

Figure 6a shows the experimental model of the material removed with different duty cycles. Figure 6b shows that the material removal depth increased linearly with the increase in the duty cycle, and the removal depth doubled by adjusting the duty cycle from 30% to 60%. Concurrently, the spatial distribution of the material removed under different duty cycles was calculated, and the linear relationship between removal efficiency and duty cycles was obtained.

**Figure 7:**
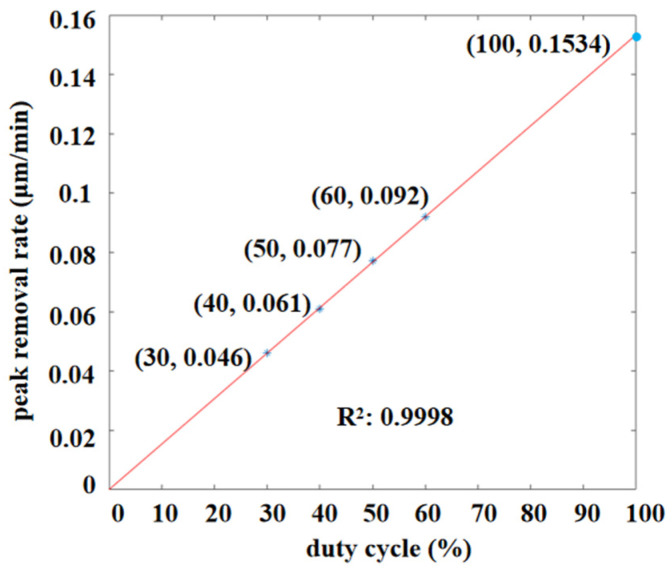
Linear relationship between duty cycle and removal efficiency.

As shown in Figure 7, the peak removal rate increased linearly with the increase in the duty cycle, with a correlation coefficient (R^2^) between the removal efficiency and duty cycle of close to one. Thus, the removal efficiency of the removal function can be controlled by adjusting the pulse duty cycle.

### 3.3. Stability Verification of the LPIB

In order to verify the application of pulsed ion beams in practical processing, it was necessary to verify the stability of the pulse. To verify the time stability of the LPIB, the material removal was measured in four consecutive 12 min segments. A 15 mm grid was selected in the experiment, the ion pulse duty cycle was kept at 25%, and the pulse frequency was kept at 100 Hz.

**Figure 8:**
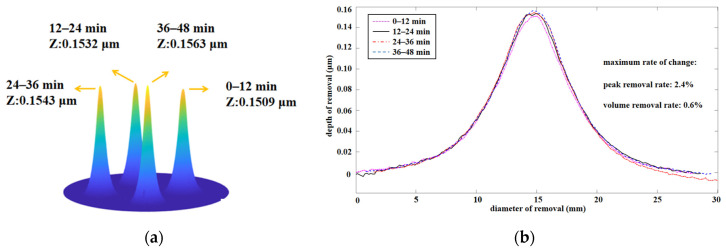
Time stability of the LPIB: (**a**) removal depth; (**b**) removal depth curve of each period.

Z in Figure 8a represents the removal depth. Figure 8b shows the maximum fluctuation of depth removal was 2.4% and the volume removal was 0.6%. Considering that the removal fluctuation of the ion source is generally within 5% [24], the experiment results show that the fluctuation of each parameter was within this range, which indicates that the time stability of the LPIB is similar to that of the normal ion beam.

## 4. Discussion

In this study, we obtained the results of the use of a pulsed ion beam by introducing a control pulsed ion beam derivation. By adjusting the pulse duty ratio and frequency, it was found to have a high material removal resolution of a single pulse, which realized a single-pulse removal depth 6.7 × 10^−4^ nm. Ultra-high resolution material-type pulsed ion beams are expected to achieve atomic-level ion beam machining material removal, which improves the accuracy when machining components.

The duty cycle of the pulse can be dynamically adjusted by the removal function. The linear relationship between the removal efficiency of the removal function and the duty cycle of the pulse was verified. Taking advantage of this nature during processing for the removal of a larger volume of materials over a larger area can remove the function of the ion beam duty ratio, increase efficiency, shorten processing time, and improve processing efficiency. At the same time, to meet the standard requirements of the desired form, it decreases the pulse duty ratio to zero, stopping the material removal and avoiding introducing residual material, which can influence surface quality. Finally, the experiment proved that LPIB has good stability and can be used in practical engineering processing.

Simultaneously, as LPIB is limited to the resolution of the measuring equipment, we adopted the method of analogical reasoning when obtaining ultra-high material removal resolution, which failed to directly measure the material removed by a single pulse. This aspect needs further improvement. In conclusion, a pulsed ion beam processing tool was proposed that can improve the removal resolution of ion beam precision materials by one to two orders of magnitude and realize the dynamic control of the removal function in the process of red ion beam processing. The innovation of the ion beam machining method is of great significance to promote the development of ultra-precision machining technology.

## 5. Conclusions

LPIB technology achieves ultra-high material thickness removal resolution at the micro scale with high macro removal efficiency. In this paper, an ion pulse output with a frequency of 100 Hz and a pulse width of 0.01 ms was stably produced through the precise control of the pulsed beam. The controllable resolution of the LPIB is 6.7 × 10^−4^ nm, which is at least one order of magnitude higher than the current low-energy ion beam. Meanwhile, LPIB has a good removal stability, and an ideal linear relationship exists between the removal rate and the pulse duty cycle. Moreover, the maximum removal resolution can be adjusted within a wide spectrum, and the removal efficiency can be dynamically adjusted. LPIB technology can be used as a new stable and predictable removal tool in cross-scale material surface treatment owing to its precise thickness and quality control. Moreover, it has various potential applications in special surface processing, ultra-precision manufacturing, adjustment of inertial device quality, ion beam sputtering deposition, and so on. It can promote the progress of ultra-precision machining technology.

## Figures and Tables

**Table 1:** Experimental parameters.

Parameter	Value	Parameter	Value
Ion energy	600 eV	Workpiece diameter	Φ100 mm
Humidity	25%	Temperature	18 (±0.2 °C)
Measurement repeatability	RMS < 0.05 nm		

## Data Availability

The data presented in this study are available on request from the corresponding author. The data are not publicly available due to the data also forming part of an ongoing study.

## References

[1] Schroeder J.B., Dieselman H.D., Douglass J.W. (1971). Technical feasibility of figuring optical surfaces by ion polishing. Appl. Opt..

[2] O’Hara A., Hannah J.R., Underwood I., Vass D.G., Holwill R.J. (1993). Mirror quality and efficiency improvements of reflective spatial light modulators by the use of dielectric coatings and chemical-mechanical polishing. Appl. Opt..

[3] Koch S.W., Knorr A. (2001). Optics in the Nano-World. Science.

[4] Chen L., Wen J., Zhang P., Yu B., Chen C., Ma T., Lu X., Kim S.H., Qian L. (2018). Nanomanufacturing of silicon surface with a single atomic layer precision via mechanochemical reactions. Nat. Commun..

[5] Hayashi S., Takano A., Takenaka H., Homma Y. (2003). SIMS study of depth profiles of delta-doped boron/silicon alternating layers by low-energy ion beams. Appl. Surf. Sci..

[6] Pachchigar V., Ranjan M., Mukherjee S. (2019). Role of Hierarchical Protrusions in Water Repellent Superhydrophobic PTFE surface produced by low energy ion beam irradiation. Sci. Rep..

[7] Shymanski V., Uglov V., Cherenda N., Pigasova V., Astashynski V., Kuzmitski A., Zhong H., Zhang S., Le X., Remnev G. (2019). Structure and phase composition of tungsten alloys modified by compression plasma flows and high-intense pulsed ion beam impacts. Appl. Surf. Sci..

[8] Cai J., Li C., Yao Y., Lyu P., Guan Q., Li Y., Lu J. (2021). Microstructural modifications and high-temperature oxidation resistance of arc ion plated NiCoCrAlYSiHf coating via high-current pulsed electron beam. Corros. Sci..

[9] Rahman T., Khan R., Qayyum H., Amin U., Ullah S., Dogar A., Mahmood A., Rafique M., Qayyum A. (2021). Characterization of microcraters fabricated on the silicon surface by single and multi-pulse laser ablation at various laser intensities. Nucl. Instrum. Methods Phys. Res. Sect. B Beam Interact. Mater. Atoms.

[10] Williams K.E., Druz B.L., Hines D.S., Londono J.F. (2001). Reactive Ion Beam Etching Method and a Thin Film Head Fabricated Using the Method. U.S. Patent.

[11] Tian Y., Zhou G., Xue S., Shi F., Song C., Li F., Zhong Y., Shen Y. (2020). Laser energy absorption prediction of silicon substrate surface from a mid- and high-spatial frequency error. Opt. Express.

[12] Pérez-Willard F., Wolde-Giorgis D., Al-Kassab T., López G.A., Mittemeijer E.J., Kirchheim R., Gerthsen D. (2008). Focused ion beam preparation of atom probe specimens containing a single crystallographically well-defined grain boundary. Micron.

[13] Zhang S., Yu X., Zhang J., Shen J., Zhong H., Liang G., Xu M., Zhang N., Ren J., Kuang S. (2021). Study of phase transformation and surface microstructure of alumina ceramic under irradiation of intense pulsed ion beam. Vacuum.

[14] Zhang W., Zhang K., Wang W., Chen Y. (2021). Detection of ion implantation in focused ion beam processing by scattering-type scanning near-field optical microscopy. Opt. Lett..

[15] Mi S., Toros A., Graziosi T., Quack N. (2019). Non-contact polishing of single crystal diamond by ion beam etching. Diam. Relat. Mater..

[16] Grobe A., Schmatz J., Littke R., Klaver J., Urai J.L. (2017). Enhanced surface flatness of vitrinite particles by broad ion beam polishing and implications for reflectance measurements. Int. J. Coal Geol..

[17] Li F., Xie X., Tie G., Hu H., Zhou L. (2016). Research on temperature field of KDP crystal under ion beam cleaning. Appl. Opt..

[18] Xiao H., Dai Y., Duan J., Tian Y., Li J. (2021). Material removal and surface evolution of single crystal silicon during ion beam polishing. Appl. Surf. Sci..

[19] Xie X., Zhou L., Dai Y., Li S. (2011). Ion beam machining error control and correction for small scale optics. Appl. Opt..

[20] Guo D., Kometani R., Warisawa S., Ishihara S. (2012). Three-dimensional nanostructure fabrication by controlling downward growth on focused-ion-beam chemical vapor deposition. Jpn. J. Appl. Phys..

[21] Tsong I.S.T., Barber D.J. (1973). Review: Sputtering mechanisms for amorphous and polycrystalline solids. J. Mater. Sci..

[22] Winters H.F. (1974). Sputtering of chemisorbed gas (nitrogen on tungsten) by low-energy ions. J. Appl. Phys..

[23] Chen Y., Zhang X. (2010). Focused ion beam technology and application in failure analysis. Proceedings of the 2010 11th International Conference on Electronic Packaging Technology & High Density Packaging.

[24] Zhou L. (2008). Study on Theory and Technology in Ionbeam Figuring for Optical Surfaces.

